# Mineral elements-mediated responses govern cadmium accumulation in plants under C14 alkane stress

**DOI:** 10.3389/fmicb.2026.1830534

**Published:** 2026-06-01

**Authors:** Lizhu Yuan, Xinzhuo Qian, Weijin Zheng, Xu Chen, Boxi Lv, Xuemei Zhong, Jonathan W. C. Wong

**Affiliations:** 1 Research Center for Eco-Environmental Engineering, Dongguan University of Technology, Dongguan, China; 2 College of Earth Sciences, Guilin University of Technology, Guilin, China

**Keywords:** Cd accumulation, mineral elements, petroleum hydrocarbon, plants growth, plants species

## Abstract

The phytoremediation of agricultural soils co-contaminated by cadmium (Cd) and petroleum hydrocarbons presents a significant challenge due to the complex interactions between pollutants. This study investigated the mechanisms governing Cd accumulation in four plant species—*Lolium perenne* L. (ryegrass), *Ricinus communis* L. (castor bean), *Amaranthus hypochondriacus* L. (amaranth), and *Mirabilis jalapa* L. (mirabilis)—under stress from C14 alkane, a model petroleum hydrocarbon. A concentration gradient of C14 alkane was applied to examine its effects on plant growth, Cd accumulation, and mineral elements uptake. Integrated multivariate analyses, including random forest analysis, mantel tests and structural equation modeling, elucidated the underlying interactions. The results demonstrated that C14 alkane stress significantly inhibited plant growth, with sensitivity varying considerably among species. Crucially, alterations in mineral elements homeostasis were identified as the central mechanism mediating plant responses. Tolerant species (ryegrass, castor bean, mirabilis) displayed a capacity for ionomic adjustment, with elements like manganese (Mn) playing a central yet complex role in modulating both stress response and Cd accumulation pathways. In contrast, the sensitive species (amaranth) failed to maintain this regulation, leading to compromised growth and Cd accumulation. This study establishes that a plant’s ionomic regulatory capacity, beyond direct C14 alkane tolerance, is a critical determinant of phytoremediation efficacy. The findings provide a novel theoretical framework for selecting remediation plants and optimizing strategies, such as targeted mineral elements management, for soils co-contaminated with heavy metals and petroleum hydrocarbons.

## 1 Introduction

Cadmium (Cd) contamination in agricultural soils has become a critical global environmental issue, primarily driven by anthropogenic activities such as industrial discharges, mining and smelting operations, and the excessive application of phosphate fertilizers (Li et al., 2021). According to the first national soil pollution survey in China, Cd is a prominent contaminant, with approximately 7.0% of the surveyed soil samples exceeding environmental quality standards. Investigations have further revealed that the average Cd concentration in soils surrounding mining areas can reach as high as 9.45 mg⋅kg^–1^ (Shi et al., 2022). As a non-biodegradable and persistent toxic heavy metal, Cd readily accumulates in soils, leading to diminished soil fertility, disruption of nutrient cycles, and adverse effects on soil microbial communities. More critically, Cd is efficiently absorbed by crops such as rice and wheat, entering the human food chain and posing serious health risks, including renal dysfunction, bone demineralization, and carcinogenic effects (Wu et al., 2023; Xie et al., 2021; Yuan et al., 2024b). Phytoremediation has emerged as a sustainable, cost-effective, and ecologically compatible alternative (Rylott and Bruce, 2022; Yuan et al., 2024a; Zou et al., 2020). Research indicates that using *Bidens pilosa* L. for remediation of Cd-contaminated soil can achieve an shoot annual Cd extraction amount of 16.8 g⋅ha^–1^ (dry weight) (Yu et al., 2022).

While earlier studies established that Chinese agricultural soils are predominantly characterized by mild to moderate Cd contamination (typically < 3 mg⋅kg^–1^) (Chen et al., 2015), which generally exerts negligible or minimal inhibitory effects on plant growth (Wang et al., 2012). A growing body of evidence indicates an increasing prevalence of co-contamination scenarios where Cd is found alongside petroleum hydrocarbons (Agnello et al., 2016; Hu et al., 2024). This emerging complexity poses significant challenges for conventional phytoremediation strategies (Zhang et al., 2026). For instance, Wang et al. (2021) demonstrated that the co-presence of pyrene substantially reduced Cd accumulation in maize, with Cd concentrations decreasing by 15–26% in roots, 32–43% in stems, and 74–81% in leaves. Furthermore, numerous studies have corroborated that petroleum hydrocarbon contamination can inhibit key physiological processes such as photosynthesis and transpiration, leading to impaired plant growth, reduced biomass yield, and even plant mortality (Duan et al., 2023).

The impact of petroleum hydrocarbons on plant mineral elements uptake constitutes a critical yet underexplored dimension. Existing studies suggest that petroleum hydrocarbons can alter soil physicochemical properties and microbial activity, thereby modulating the bioavailability of essential mineral elements such as nitrogen, phosphorus, and potassium (Haider et al., 2021). On one hand, mineral elements may alleviate hydrocarbon-induced oxidative stress and enhance plant tolerance, supporting biomass accumulation (Yuan et al., 2022)—a vital trait for phytoremediation efficacy, particularly in species reliant on high biomass for metal extraction (Yu et al., 2022). On the other hand, shifts in mineral elements acquisition may indirectly influence Cd uptake and translocation, given that Cd competes with nutrients like calcium, zinc, and iron due to similar charge and ionic radius (Huang et al., 2020). As plant species vary in their tolerance to petroleum hydrocarbons, the hydrocarbon-induced modulation of mineral elements uptake and its subsequent effect on Cd accumulation likely differ substantially among species. Thus, how petroleum hydrocarbons affect mineral elements absorption in different plants and thereby regulate Cd accumulation remains an open question.

In the present study, the impact of petroleum hydrocarbons on Cd phytoremediation was investigated using four distinct plant species: *Lolium perenne* L. (ryegrass), *Ricinus communis* L. (castor bean), *Amaranthus hypochondriacus* L. (amaranth), and *Mirabilis jalapa* L. (mirabilis). All four species have demonstrated significant potential for Cd remediation (Li et al., 2020; Li Y. et al., 2025; Lu et al., 2025; Ruiz Olivares et al., 2013). While ryegrass and mirabilis are known to exhibit high tolerance to petroleum hydrocarbons (Li Y. et al., 2025; Liu et al., 2023), the tolerance levels of castor bean and amaranth to such contaminants remain unclear. Short-chain alkanes (C_10_–C_20_) are readily absorbed by plants and can induce cytotoxic effects at the cellular level, posing a particular threat to plant development (Proietto et al., 2024; Yuan et al., 2022). However, whereas extensive research has focused on total petroleum hydrocarbons and polycyclic aromatic hydrocarbons (Liu et al., 2023; Wang et al., 2021), the specific influence of short-chain alkanes on phytoremediation efficiency in co-contaminated soils remains poorly understood. To address this knowledge gap, C14 alkane was selected as a representative short-chain petroleum hydrocarbon. The objectives were: (1) to investigate the effects of C14 alkane on plant growth, Cd accumulation, and mineral element uptake, (2) to elucidate the mineral elements mediated mechanisms underlying Cd accumulation under C14 alkane stress using random forest, Mantel tests, and structural equation modeling, and (3) to identify species-specific ionomic regulatory strategies. This study is significant because it moves beyond merely documenting the inhibitory effects of organic pollutants on metal uptake. Instead, it reveals that a plant’s ionic regulatory capacity—its ability to maintain mineral element homeostasis under stress—is a critical determinant of phytoremediation efficacy in co-contaminated soils. Our findings provide a novel theoretical framework for selecting remediation plants and optimizing nutrient management strategies for soils co-contaminated with heavy metals and petroleum hydrocarbons.

## 2 Materials and methods

### 2.1 Chemicals and soil

Seeds of four potential Cd and petroleum-remediating plant species—ryegrass, castor bean, amaranth, and mirabilis—were purchased from Suqian Sunrise Seed Industry Co., Ltd (China). All other chemicals, of analytical grade or higher purity, were obtained from Sinopharm Chemical Reagent Co., Ltd (China), while standard solutions for Cd and mineral nutrients were sourced from the National Reference Material (RM) Resources Network. Ultrapure water (Milli-Q IQ 7000, Millipore, France) was used for the preparation of all aqueous solutions. The clean soil was collected and characterized, and the main physicochemical properties of were presented in Supplementary Table 1. The Cd-C14 alkane co-contaminated soil was prepared following procedures identical to those described in our previous study (Yuan et al., 2022). Cd-contaminated soil was first prepared by mixing soil with an aqueous cadmium nitrate solution (soil: solution = 1:1, m/V) and incubating at room temperature for 1 month. After air-drying and sieving (0.84 mm), this Cd-spiked soil was mixed with C14 alkane (dissolved in n-hexane) at defined volume-to-mass ratios (0.1–2.0 mL g^–1^). The mixture was then agitated daily for 2 weeks in a fume hood to ensure uniform distribution and complete solvent evaporation, yielding the Cd-C14 alkane co-contaminated soil.

### 2.2 Experiments setup

The soil in the Cd-C14 alkane co-contamination treatments was uniformly spiked with Cd at a concentration of 2 mg⋅kg^–1^, representing a typical moderate Cd contamination level in Chinese agricultural soils (Chen et al., 2015). Six different C14 alkane levels were established, expressed as the volume (mL) to soil mass (g) ratio: 0% (control, CK), 0.1, 0.2, 0.5, 1.0, and 2.0%. Each of the four plant species—ryegrass, castor bean, amaranth, and mirabilis—were cultivated in these treated soils. All treatments were conducted in triplicate.

The experiment was conducted in rectangular containers measuring 22 cm (length) × 16 cm (width) × 12.5 cm (height). Each container was filled with approximately 4.5 kg of air-dry contaminated soil. Prior to sowing, the soil was irrigated with tap water until leaching occurred, ensuring complete wetting. Seeds were surface-sterilized with 30% (v/v) H_2_O_2_ for 30 min, rinsed thoroughly with ultrapure water, and then sown when soil moisture content reached approximately 18%. Ryegrass was sown uniformly in six rows per container, with 2.0 g of seeds per row. For castor bean, amaranth, and mirabilis, the same sowing method was applied, with three rows per container, each containing two evenly spaced holes. Two seeds were dibbled per hole, totaling 12 seeds per container. After germination, seedlings were thinned to one plant per hole at the 2-week stage, resulting in six individual plants per container. The plants were grown under artificial illumination provided by fluorescent lamps, with an average light intensity of 3,000 lux and a photoperiod of 16/8 h (light/dark) regulated by an automatic timer. The growth chamber temperature was maintained at 24–26°C during the light period and 18–20°C during the dark period. Containers were watered every other day to maintain soil moisture. No fertilizer was applied throughout the experiment to avoid confounding nutrient effects.

After 60 days of growth, plant roots and shoots were separately harvested. Fresh plant tissues were rinsed with ultrapure water, followed by purified water, and gently blotted dry. To remove surface-adhered metal ions, roots were immersed in 20 mM Na_2_EDTA for 15 min and then rinsed three times with ultrapure water. The samples were subsequently heated at 105°C for 20 min and dried to constant weight at 70°C. Dry biomass was recorded for each sample.

### 2.3 Analytical method

Soil pH was determined in a 1:2.5 (w/v) soil-to-water slurry with a pH meter (S220 Seven Compact, Mettler-Toledo International Inc., Switzerland). Bioavailable Cd and Mn in soil were extracted with a DTPA solution containing 5 mmol⋅L^–1^ diethylenetriaminepentaacetic acid, 10 mmol⋅L^–1^ triethanolamine, and 10 mmol⋅L^–1^ CaCl_2_, adjusted to pH 7.3. Plant shoot and root samples were digested with a mixture of HNO_3_ and HClO_4_ (Yuan et al., 2021). The resulting digests and DTPA extracts were filtered through a 0.22 μm membrane and analyzed for metal concentrations using Inductively Coupled Plasma Mass Spectrometer (ICP-MS, iCAP RQ, Thermo Fisher Scientific, United States).

For C14 alkane analysis, soil samples were extracted in triplicate by repeated shaking and ultrasonication (30 min per cycle) using a dichloromethane-acetone mixture (1:1, v/v). The combined extract was concentrated, reconstituted in 5.0 mL of n-hexane, and filtered through a 0.22 μm membrane. Analysis was performed on a gas chromatograph equipped with a flame ionization detector (GC-FID, Ultra-ISQ, Thermo Fisher Scientific, United States). High-purity nitrogen was used as the carrier gas at a flow rate of 1.0 mL⋅min^–1^. The injector and detector temperatures were set to 250 and 300°C, respectively. The oven temperature program started at 60°C, increased to 290°C at 15°C⋅min^–1^, and held for 5 min.

### 2.4 Quality assurance vs. quality control

Quality assurance and quality control procedures were implemented to ensure data reliability. Reagent blanks were used to correct instrument readings. A plant standard reference material (GBW07602) was included in each digestion and analysis batch, yielding a mean recovery of 108.7% ± 5.6%. All samples were analyzed in triplicate, and a standard was run after every 20 samples to monitor instrument drift.

### 2.5 Statistical analyses

The bioconcentration factor (BCF), translocation factor (TF), Cd uptake amount (Up) in roots and shoots were calculated according to the following (Equations 1–3):


B⁢C⁢F=C⁢p/C⁢s (1)

T⁢F=C⁢p/C⁢r (2)

U⁢p=C⁢p×M⁢p (3)

where Cp and Cr represents the Cd concentration (mg⋅ kg^–1^) in shoot and root, respectively. Cs denotes the Cd concentration in soil (mg⋅ kg^–1^), Mp is the dry biomass of plant aboveground part (kg per pot), respectively.

All data are presented as mean ± standard deviation (SD). Statistical analyses were performed using SPSS (IBM SPSS Statistics 22, New York, United States). One-way analysis of variance (ANOVA) was applied to assess differences among treatments, with a significance level set at *p* < 0.05. Bar graphs were generated using Origin software (version 8.0, Origin Lab Corporation, Northampton, United States). Random forest (RF) analysis for variable importance, Mantel tests, and structural equation modeling (SEM) were conducted in R (v4.3.1) using the randomForest, linkET, and piecewiseSEM packages, respectively.

## 3 Results

### 3.1 Plant biomass

**FIGURE 1:**
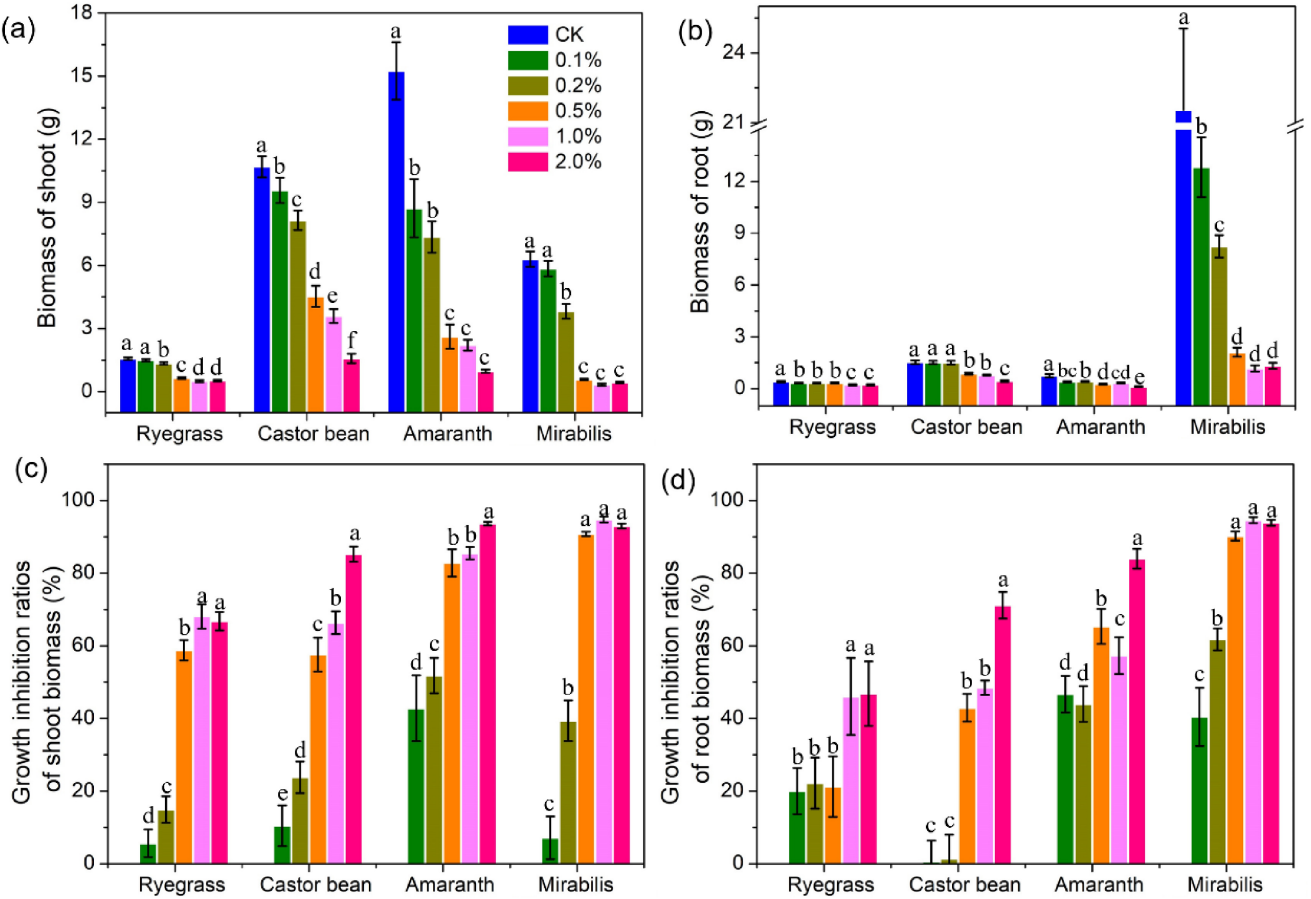
Effect of various C14 alkane levels on the biomass and growth inhibition ratios of different plants, **(a)** biomass of shoot, **(b)** biomass of root, **(c)** growth inhibition ratios of shoot, **(d)** growth inhibition ratios of root. Each value represents the mean ± standard deviation of three independent experiments. Different lowercase letters on the column represented significant difference in different treatments of C14 alkane concentration (*P* < 0.05).

Increasing C14 alkane concentration induced a concentration-dependent decrease in both shoot and root biomass across all four plant species, with a concomitant rise in growth inhibition rates (Figure 1). The phytotoxic effect of C14 alkane was highly species-specific. At lower stress levels (0.1% C14 alkane), ryegrass, castor bean, and mirabilis exhibited moderate sensitivity, with shoot inhibition rates ranging from 5.63 to 10.47%. In contrast, amaranth was substantially more affected, showing a shoot inhibition rate of 42.82% even at the same low concentration. A marked toxicity threshold was observed at 0.5% C14 alkane, where inhibition rates increased sharply across all species. Under the highest stress treatment (2.0% C14 alkane), ryegrass demonstrated the strongest tolerance with a shoot inhibition rate of 66.77%, while the other three species experienced severe growth suppression, each exceeding 85% inhibition. A similar concentration-dependent inhibitory pattern was observed for root biomass across all species (Figures 1b,d). These results clearly indicate that ryegrass possesses the highest tolerance to C14 alkane, whereas amaranth is the most sensitive species under the tested conditions.

### 3.2 Cd accumulation in plants

**FIGURE 2:**
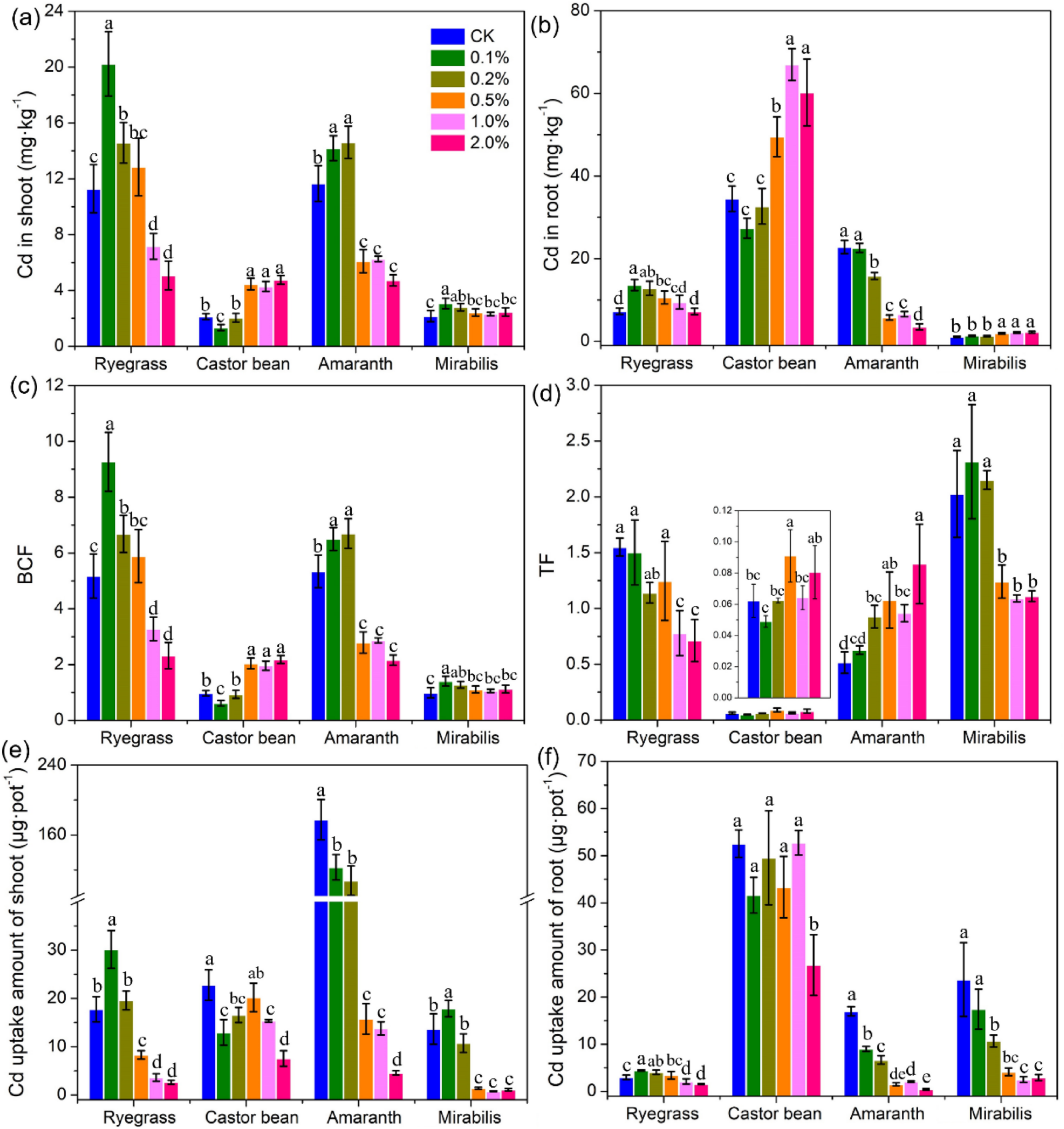
Effect of various C14 alkane levels on Cd concentration, BCF, TF and Cd uptake amounts of different plants, **(a)** Cd concentration in shoot, **(b)** Cd concentration in root, **(c)** BCF, **(d)** TF, **(e)** Cd uptake amount in shoot, **(f)** Cd uptake amount in root. BCF refers to the bioconcentration factors of Cd, TF refers to the translocation factors of Cd. Each value represents the mean ± standard deviation of three independent experiments. Different lowercase letters on the column represented significant difference in different treatments of C14 alkane concentration (*P* < 0.05).

C14 alkane stress significantly altered Cd distribution and accumulation, revealing complex, species-dependent responses (Figure 2). In ryegrass, Cd concentrations in both roots and shoots initially increased and then decreased with increasing C14 alkane, peaking at 13.61 and 20.22 mg⋅kg^–1^, respectively, at the 0.1% treatment. Castor bean displayed an opposite trend, with root and shoot Cd concentrations reaching minima at 0.1% C14 alkane (27.39 and 1.35 mg⋅kg^–1^) before increasing progressively. For amaranth, rising C14 alkane concentrations significantly reduced root Cd content, while low concentrations (0.1 and 0.2%) increased shoot Cd content, which was then suppressed at higher levels. Mirabilis showed a different response, with C14 alkane treatment generally enhancing Cd content in both its roots and shoots across the concentration gradient.

The influence of C14 alkane on the BCF of the four plant species (Figure 2c) exhibited a pattern similar to its effect on shoot Cd concentration. Under the 0.1% C14 alkane treatment, ryegrass showed the highest BCF value of 9.27, followed by amaranth with a BCF of 6.70 at 0.2% C14 alkane. Notably, most treatments resulted in BCF > 1, except for castor bean at low stress (0.1 and 0.2%) and mirabilis in the CK, indicating a general enhancement of Cd phytoextraction potential under mild to moderate C14 alkane stress. With the exception of the CK, castor bean under 0.1 and 0.2% C14 alkane treatments, as well as mirabilis in CK, all other treatments resulted in BCF values > 1. Regarding the TF (Figure 2d), increasing C14 alkane concentration led to a gradual decrease in ryegrass but a progressive increase in amaranth. Significant reductions in TF were observed for mirabilis at and above the 0.5% C14 alkane concentration. In contrast, castor bean generally maintained low TF values across treatments, with no clear trend detected.

As shown in Figures 2e,f, the highest Cd uptake of shoot was observed in amaranth under the CK treatment, reaching 177.6 μg⋅pot^–1^. With increasing C14 alkane concentration, shoot Cd uptake in amaranth progressively decreased, which plummeted by over 97 under 2% C14 alkane stress, highlighting its extreme sensitivity. Ryegrass and mirabilis exhibited similar trends in shoot Cd uptake, which increased initially and then gradually declined with rising C14 alkane levels. The maximum uptake values for ryegrass and mirabilis shoots were recorded at 0.1% C14 alkane, measuring 30.17 and 17.90 μg⋅pot^–1^, respectively. In castor bean, all C14 alkane treatments reduced shoot Cd uptake compared to the CK. Interestingly, the inhibitory effect weakened as the concentration increased from 0.1 to 0.5%, with Cd uptake reaching 20.21 μg⋅pot^–1^ at 0.5%—close to the CK level. However, a further increase to 2% C14 alkane led to a significant reduction in shoot Cd uptake. In ryegrass, root Cd uptake followed a trend similar to that of its shoots. For castor bean, root Cd uptake remained largely unaffected by most C14 alkane treatments, except for a significant reduction observed at the 2% level. In contrast, both amaranth and mirabilis exhibited a gradual decrease in root Cd uptake with increasing C14 alkane concentration.

### 3.3 C14 alkane in soil

**FIGURE 3:**
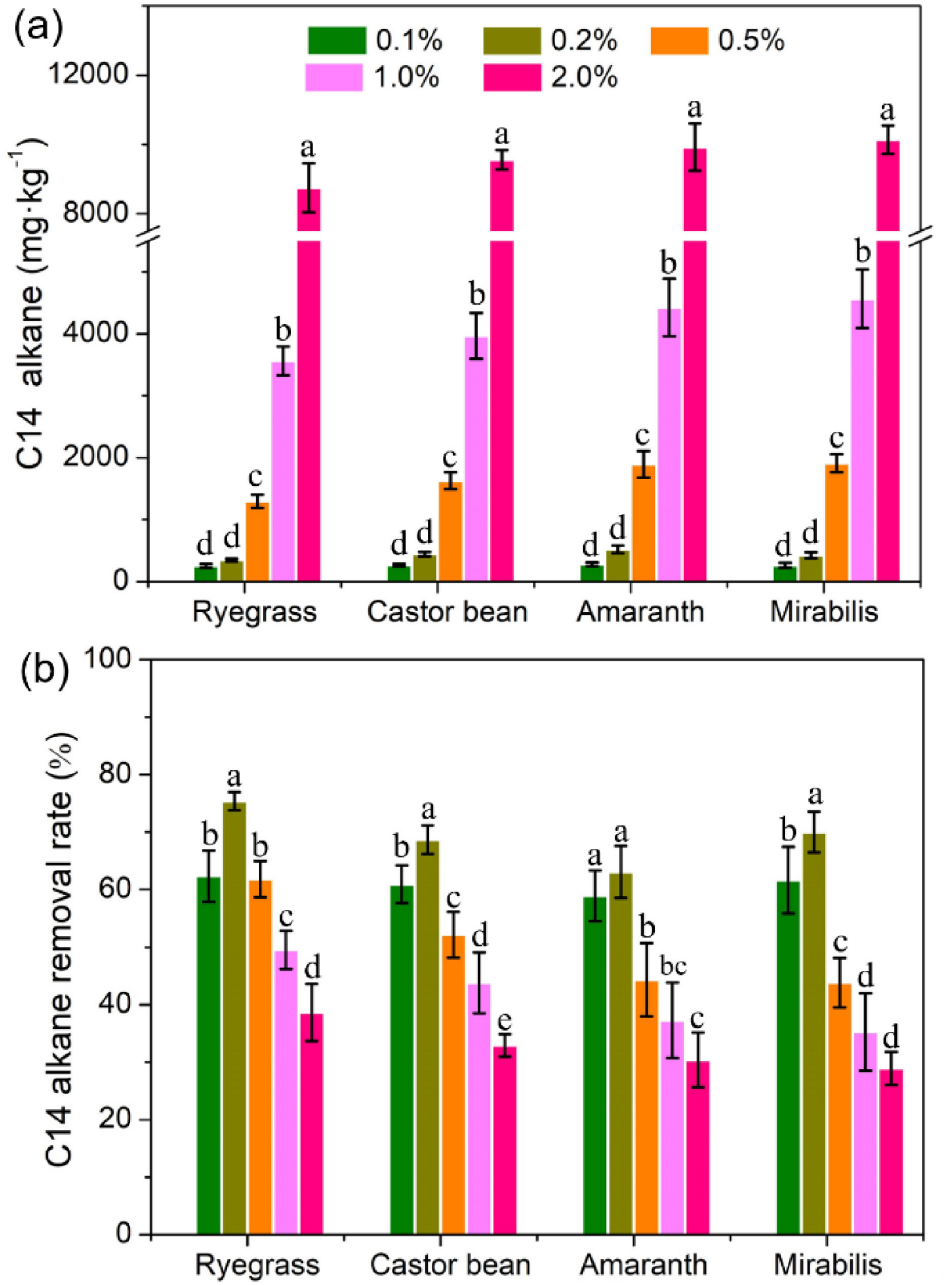
The residual concentration and removal rate of C14 alkane in soil after different treatments. **(a)** Residual concentration of C14 alkane, **(b)** removal rate of C14 alkane. Each value represents the mean ± standard deviation of three independent experiments. Different lowercase letters on the column represented significant difference in different treatments of C14 alkane concentration (*P* < 0.05).

The initial C14 alkane concentrations in the spiked soils were 681.7, 1412.5, 3401.6, 7059.1, and 14262.3 mg⋅kg^−1^, corresponding to the preparation levels of 0.1, 0.2, 0.5, 1.0, and 2.0% (v/w), respectively. After 60 days of plant cultivation, the residual concentration of C14 alkane in soil decreased significantly across all treatments. Due to the gradient in initial C14 alkane concentrations, which ranged from 0.1 to 2%, the residual levels increased correspondingly as the initial spiking concentration increased. The removal efficiency of C14 alkane showed a consistent trend: it initially increased and then gradually declined. The highest removal rates, ranging between 63.08 and 75.41%, were achieved at an initial C14 alkane concentration of 0.2% (Figure 3).

### 3.4 Soil pH, available Cd and Mn

**FIGURE 4:**
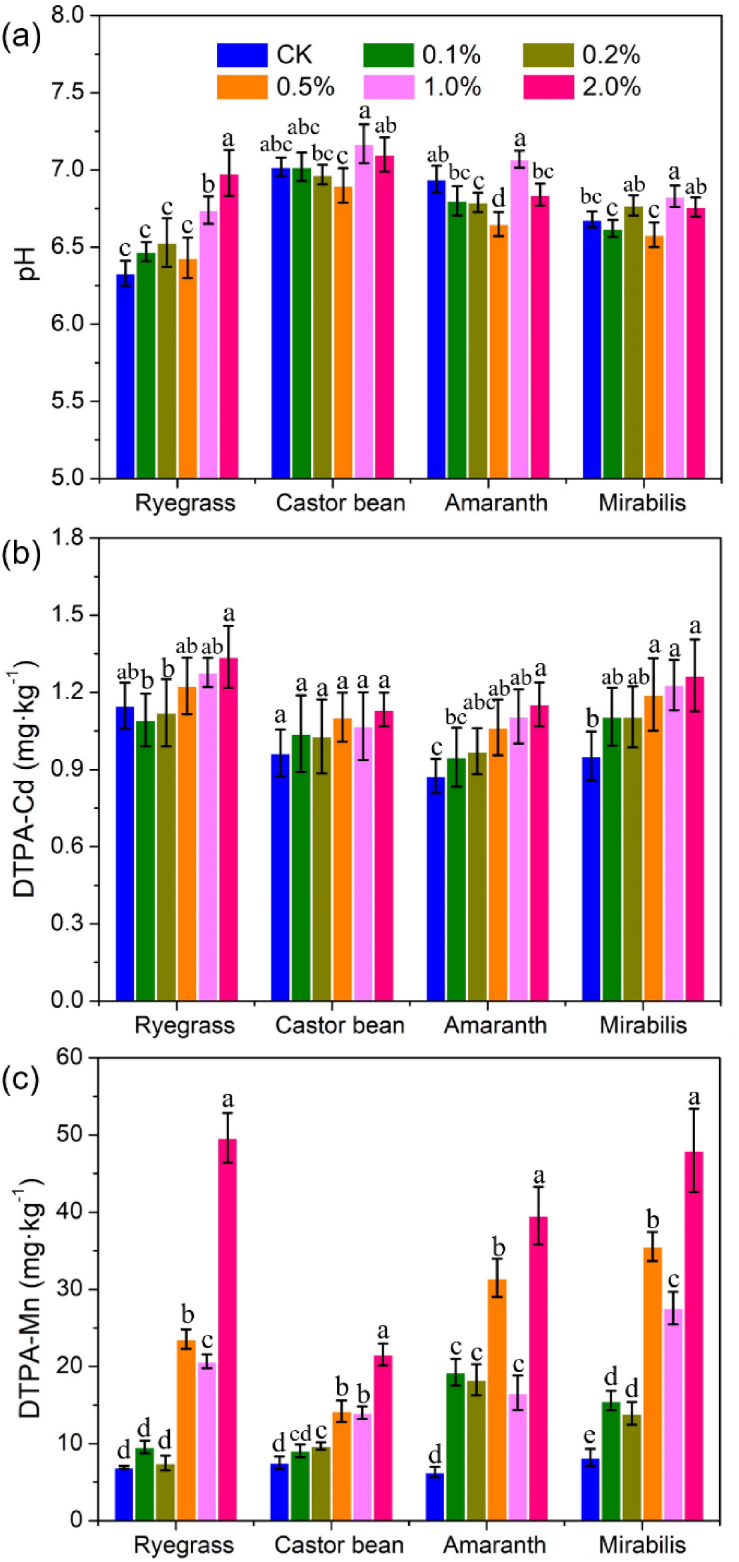
Soil pH, bioavailable Cd and Mn concentration in soil after different treatments. **(a)** soil pH, **(b)** bioavailable Cd concentration, **(c)** bioavailable Mn concentration. DTPA_Cd and DTPA_Mn represent the concentration of bioavailable Cd and Mn in soil, respectively. Each value represents the mean ± standard deviation of three independent experiments. Different lowercase letters on the column represented significant difference in different treatments of C14 alkane concentration (*P* < 0.05).

Soil pH exhibited minimal variation across all treatments, ranging from 6.33 to 7.17 (Figure 4a). In the ryegrass group, the addition of 1 and 2% C14 alkane significantly increased soil pH compared to the treatment without C14 alkane. For amaranth, soil pH was significantly reduced under the 0.2 and 0.5% C14 alkane treatments. In the mirabilis group, only the 1% C14 alkane treatment caused a significant increase in soil pH. No significant differences in soil pH were detected between other C14 alkane treatments and their corresponding CK across all four plant species.

Bioavailable Cd and Mn were determined as DTPA-extractable fractions. As shown in Figure 4b, in the ryegrass group, only the 2% C14 alkane treatment resulted in a significant increase in bioavailable Cd compared to the CK, while no significant differences were observed at other C14 alkane concentrations. In the castor bean group, bioavailable Cd levels showed no significant differences across all C14 alkane treatments. In contrast, for both amaranth and mirabilis groups, bioavailable Cd content increased progressively with increasing C14 alkane concentration. For bioavailable Mn (Figure 4c), all C14 alkane treatments significantly enhanced its concentrations, except for ryegrass under 0.1 and 0.2% C14 alkane and castor bean under 0.1% C14 alkane. Notably, at the 2% C14 alkane level, bioavailable Mn increased to 2.9–7.2 times that of the CK treatments.

### 3.5 Mineral elements in plants

The concentrations of mineral elements (K, Ca, Mg, Fe, Mn, Cu, and Zn) in plant roots and shoots are shown in Supplementary Figure 1. Application of C14 alkane differentially influenced the concentrations of various nutrients in the roots and shoots of the four plant species. Notably, C14 alkane treatment significantly enhanced Mn concentrations in both roots and shoots across all species. For ryegrass, castor bean, and mirabilis, tissue Mn concentrations increased progressively with rising C14 alkane levels. In contrast, amaranth exhibited a gradual decrease in Mn concentration with increasing C14 alkane, though levels remained significantly higher than those in the CK.

All C14 alkane treatments significantly reduced Mg and Fe concentrations in ryegrass shoots, while increasing Fe and Zn concentrations in its roots. Additionally, Mg and Ca concentrations in ryegrass roots were elevated by C14 alkane application, with the exception of the 2% treatment. In castor bean, all C14 alkane levels led to a significant decrease in shoot Mg concentration. For amaranth, C14 alkane addition significantly increased K concentration in the shoots but decreased it in the roots. Meanwhile, in mirabilis, significant increases in shoot K and Cu concentrations were induced by C14 alkane treatments.

### 3.6 Influence of environmental factors on Cd accumulation

**FIGURE 5:**
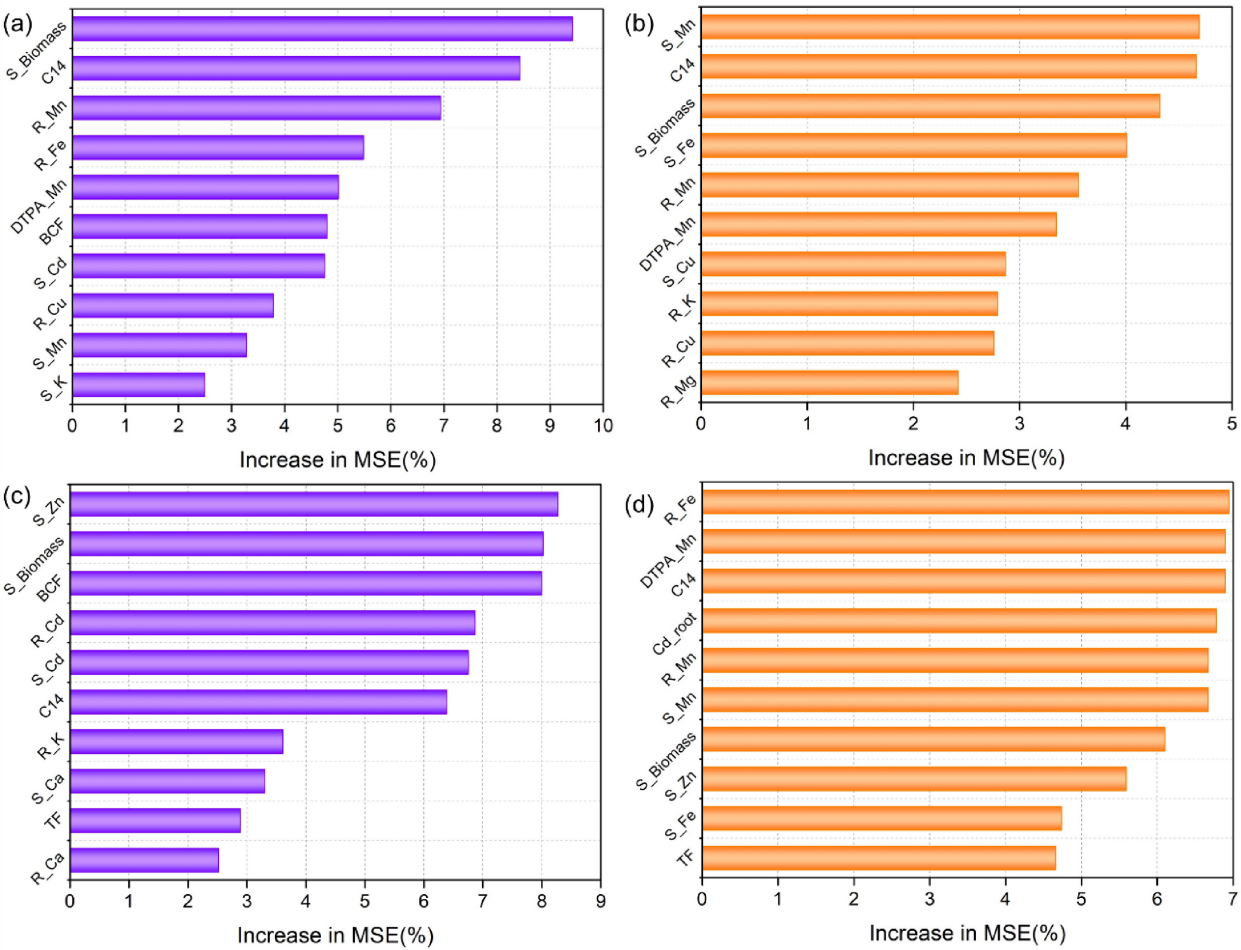
The importance ranking diagram of soil environmental factors and plant factors affecting Cd accumulation in ryegrass **(a)**, castor bean **(b)**, amaranth **(c)**, and mirabilis **(d)**. S_M and R_M denote the metal M concentration in the shoot and root of the plant, respectively. C14 denotes the residual concentration of C14 alkane in soil. DTPA_Mn represents the concentration of bioavailable Mn in soil. S_Biomass refers to the shoot dry biomass. BCF refers to the bioconcentration factors of Cd, TF refers to the translocation factors of Cd.

**FIGURE 6:**
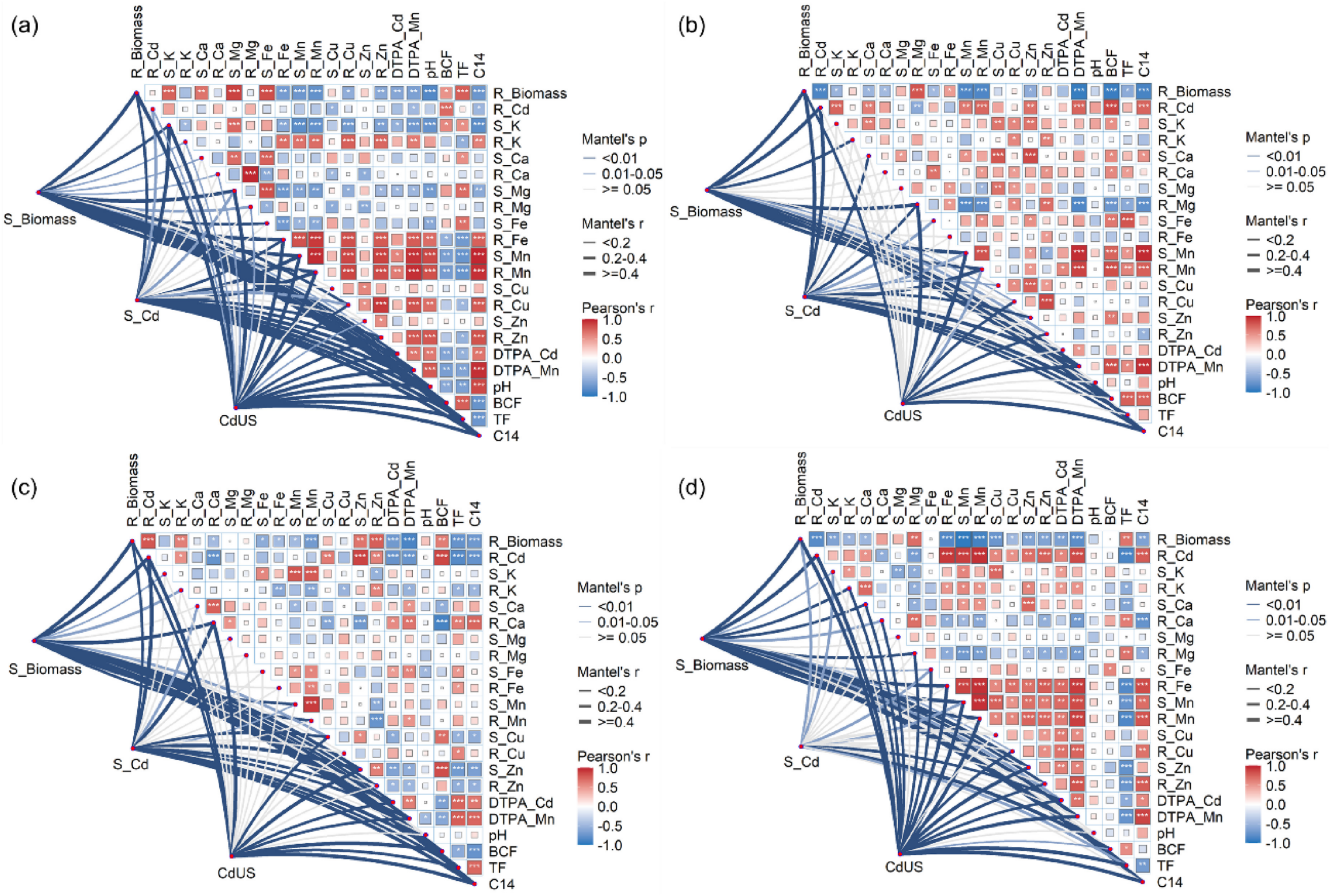
Correlations of soil and plant factors with Cd accumulation for ryegrass **(a)**, castor bean **(b)**, amaranth **(c)**, and mirabilis **(d)**. Edge width corresponds to Mantel’s r value and the edge color denotes statistical significance. Pairwise correlations of soil and plant factors were shown with a color gradient denoting Pearson’s correlation coefficient. S_M and R_M denote the metal M concentration in the shoot and root of the plant, respectively. C14 denotes the residual concentration of C14 alkane in soil. DTPA_Cd and DTPA_Mn represent the concentrations of bioavailable Cd and Mn in soil, respectively. S_Biomass and R_Biomass refer to the shoot and root dry biomass, respectively. BCF refers to the bioconcentration factors of Cd, TF refers to the translocation factors of Cd. CdUS indicates the Cd uptake in plant shoots.

**FIGURE 7:**
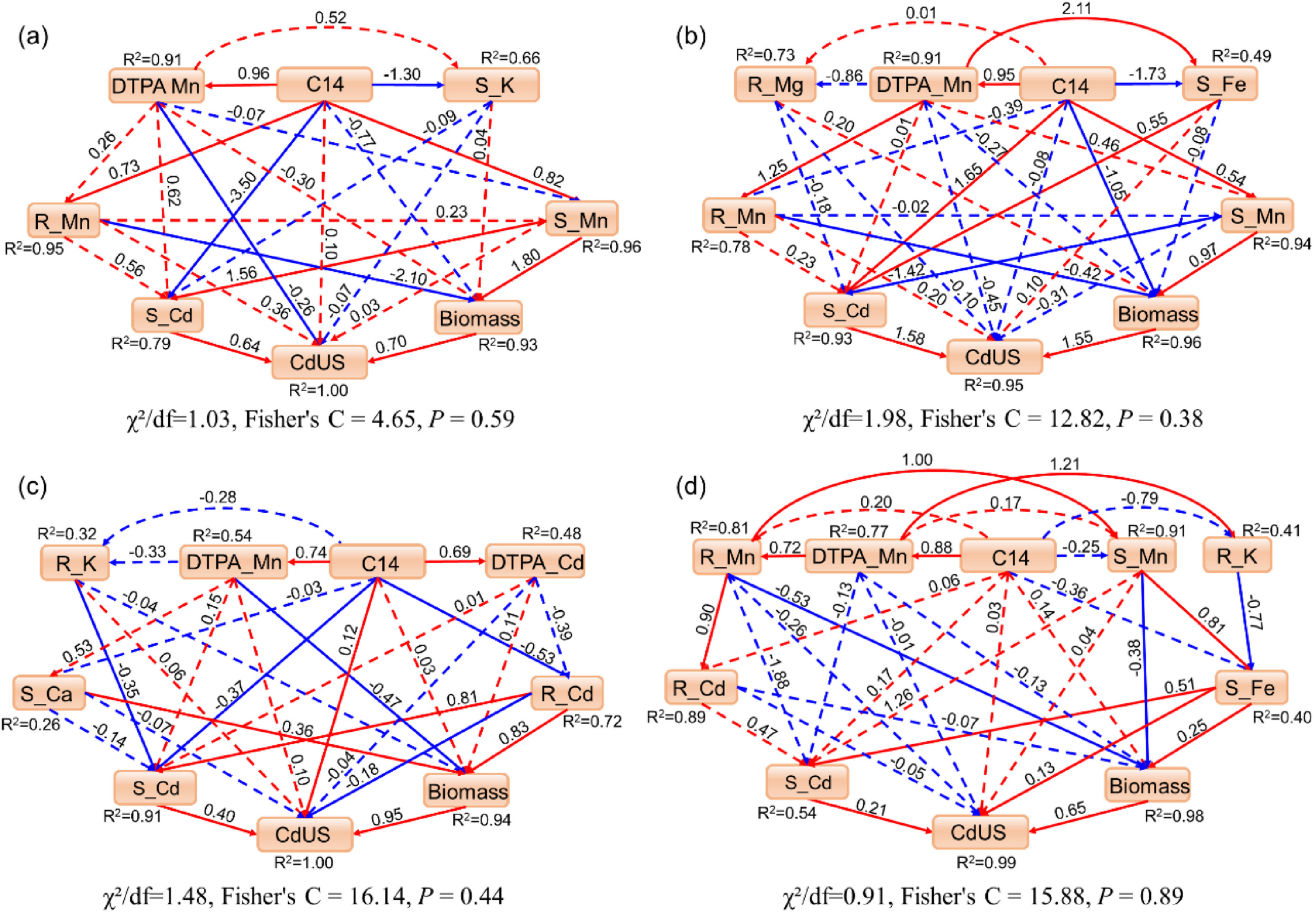
SEM of the effects of soil and plant factors on the Cd accumulation in ryegrass **(a)**, castor bean **(b)**, amaranth **(c)**, and mirabilis **(d)**. Red and blue arrows correspond positive and negative relationships, respectively. The solid line and short dash line indicate significant (*P* < 0.05) and non-significant (*P* > 0.05) relationships, respectively. Numbers adjacent to arrows are standardized path coefficients, analogous to partial regression weights and indicative of the effect size of the relationship. *R*^2^ denotes the proportion of variance explained and appears below every response variable in the model. S_M and R_M denote the metal M concentration in the shoot and root of the plant, respectively. C14 denotes the residual concentration of C14 alkane in soil. DTPA_Cd and DTPA_Mn represent the concentrations of bioavailable Cd and Mn in soil, respectively. Biomass refers to the shoot dry biomass. CdUS indicates the Cd uptake in plant shoots.

To elucidate the potential drivers governing Cd accumulation in the four plant species, we performed random forest analysis to evaluate the relative importance of soil and plant factors. Further, Mantel tests and SEM were employed to examine the linkages between plant Cd accumulation and key soil or plant parameters (Figures 5–7). The random forest analysis (Figure 5) revealed that shoot biomass was the strongest predictor of Cd accumulation in ryegrass, followed by residual C14 alkane concentration in soil and root Mn content (R_Mn). For castor bean, shoot Mn content (S_Mn) emerged as the most important predictor, followed by residual C14 alkane concentration and shoot biomass. In amaranth, shoot Zn content (S_Zn) was identified as the strongest predictor, followed by its biomass and BCF. For mirabilis, root Fe content (R_Fe) was the top predictor, followed by soil bioavailable Mn and residual C14 alkane concentration.

Mantel tests (Figure 6a) showed that residual C14 alkane, pH, bioavailable Mn and Cd, root Mn, and shoot Mn and Fe were significantly negatively correlated with shoot biomass, Cd concentration, and Cd uptake in ryegrass, while root K, Cu and Zn were also negatively correlated with shoot biomass. Conversely, root biomass, shoot K, BCF, and TF were positively correlated with these parameters, moreover, shoot Ca, Mg, Fe positively correlated with shoot biomass, and root Cd positively correlated with shoot Cd and total Cd uptake. For castor bean (Figure 6b), residual C14 alkane, bioavailable Mn, root Cd, Mn, shoot Mn, K, Ca, Zn, BCF, and TF were negatively correlated with shoot biomass but positively correlated with shoot Cd concentration. In contrast, root biomass and root Fe, Mg showed positive correlations with shoot biomass but negative correlations with shoot Cd concentration. Cd uptake was negatively associated with residual C14 alkane, bioavailable Mn, and shoot Mn, yet positively correlated with root biomass, shoot Mg, and root Mg, Cu, Zn. In amaranth (Figure 6c), residual C14 alkane, bioavailable Mn and Cd, root Ca and TF were negatively correlated with shoot biomass, Cd concentration, and Cd uptake, whereas root biomass, root Cd, and shoot Zn were positively correlated with these parameters. Additionally, root Mn and shoot Fe showed negative correlations with both shoot biomass and Cd uptake, while root K and Zn exhibited positive correlations with them. For mirabilis (Figure 6d), residual C14 alkane, bioavailable Mn and Cd, root Cd, Mn, K, Fe, Zn, and shoot Mn, Ca, Zn were negatively correlated with shoot biomass and Cd uptake. In contrast, root biomass, root Ca and Mg, and TF showed positive correlations with these parameters. Additionally, shoot K and Cu and root Cu were negatively correlated with shoot biomass.

SEM results (Figure 7) revealed species-specific causal pathways. In ryegrass and castor bean, residual C14 alkane had no direct effect on Cd uptake but significantly increased soil bioavailable Mn, which in turn modulated Cd accumulation and biomass. Specifically, in ryegrass, shoot Mn content exerted significant positive direct effects on both shoot Cd concentration (β = 1.56) and shoot biomass (β = 1.80). In castor bean, shoot Mn content showed a significant negative direct effect on shoot Cd (β = –1.42) but a positive effect on shoot biomass (β = 0.97). In amaranth, residual C14 alkane directly increased Cd uptake (β = 0.12) and soil bioavailable Mn and Cd, while negatively affecting root and shoot Cd (β = −0.53 and −0.37, respectively). Soil bioavailable Mn reduced shoot biomass (β = −0.47). In mirabilis, residual C14 alkane only increased soil bioavailable Mn (β = 0.88), with root and shoot Mn suppressing shoot biomass (β = −0.53 and −0.38, respectively), whereas shoot Fe enhanced shoot biomass (β = 0.25), shoot Cd concentration (β = 0.51), and Cd uptake (β = 0.13).

## 4 Discussion

### 4.1 Differential effects of C14 alkane stress on mineral elements uptake in different plant species

C14 alkane stress markedly altered plant uptake of mineral elements (Supplementary Figure 1), primarily through the following mechanisms. First, C14 alkane stress influences the speciation of mineral elements in soil. Petroleum hydrocarbons have been shown to significantly affect soil physicochemical properties and modify microbial community structure (Haider et al., 2021), thereby influencing the bioavailability of mineral elements and subsequently their uptake by plants. Our results demonstrate that C14 alkane stress significantly affected soil pH and the content of bioavailable Mn (Figure 4). Second, C14 alkane stress likely modulates mineral elements acquisition by impacting root activity (Giehl et al., 2023). In a hydroponic experiment, Xia et al. (2017) reported that low doses of bisphenol A (1.5 mg⋅L^–1^) can promote root vitality and enhance the uptake of P, K, Mg, and Ca, whereas high doses (6.0, 12.0 mg⋅L^–1^) inhibit root activity and reduce the absorption of P, K, and Mg. C14 alkane stress may exert analogous effects on plant root systems. Concurrently, C14 alkane stress induces membrane lipid peroxidation in plants (Yuan et al., 2022), increasing malondialdehyde content and electrolyte leakage. This disruption of cell membrane integrity and impairment of ion channel function consequently interfere with transmembrane transport of mineral elements (Minami et al., 2025; Zeng et al., 2020). Furthermore, plants possess intrinsic ion homeostasis mechanisms. For instance, to mitigate Cd toxicity, plants can actively increase the uptake of Ca and Mg to stabilize cell wall structure, maintain enzymatic activity, and reduce Cd-induced damage to the photosynthetic apparatus (Zeng et al., 2020).

The direction and magnitude of C14 alkane’s impact on mineral elements uptake exhibited significant species-dependent variation, likely attributable to interspecific differences in C14 alkane tolerance. Previous research has identified ryegrass and mirabilis as species with considerable proficiency for petroleum hydrocarbon remediation (Li Y. et al., 2025; Liu et al., 2023), suggesting potentially higher inherent tolerance to C14 alkane. Consistent with this, in ryegrass and mirabilis, the concentrations of most mineral elements in roots generally exhibited increasing or initial increase followed by decrease trends with rising C14 alkane concentration. This response pattern may be linked to C14 alkane-induced stimulation of root activity (Xia et al., 2017) or the activation of intrinsic ion regulatory mechanisms to counteract C14 alkane toxicity (Zeng et al., 2020). In contrast, castor bean and amaranth are presumably less tolerant to C14 alkane. In these species, root concentrations of elements such as Fe and Mg (in castor bean) and K and Zn (in amaranth) declined progressively with increasing C14 alkane levels, whereas other elements showed no clear trends. This suppression of uptake likely stems from C14 alkane-induced inhibition of root activity (Xia et al., 2017) and disruption of cell membrane integrity, which interferes with transmembrane ion transport (Zeng et al., 2020).

### 4.2 Mechanisms of mineral elements-mediated Cd accumulation in plants under C14 alkane stress

Plant capacity for Cd accumulation is governed by both biomass and tissue Cd concentration. Biomass and growth inhibition rate results (Figure 1) confirmed that C14 alkane stress significantly suppressed the growth of all four plant species. Notably, amaranth exhibited a significantly higher growth inhibition rate under 0.1–1% C14 alkane stress compared to the other three species. This growth suppression can be largely attributed to the disruptive impact of C14 alkane on fundamental plant physiological processes. Consistent with previous findings, petroleum hydrocarbons are known to impair photosynthetic efficiency, leading to suppressed plant development (Haider et al., 2021). For instance, Duan et al. (2023) reported that *Hippophae rhamnoides* exposed to soil contaminated with 20 g kg^–1^ petroleum hydrocarbons showed an approximately 87% reduction in shoot biomass and a 60% decrease in chlorophyll and carotenoid content compared to the control. In parallel, C14 alkane stress significantly altered plant Cd uptake (Figure 2). The Cd concentrations in the shoots of ryegrass, amaranth, and mirabilis initially increased and then gradually decreased with increasing C14 alkane concentration, a trend consistent with previous findings on the effect of pyrene on Cd uptake in maize (Zhang et al., 2009). In contrast, the response of shoot Cd concentration in castor bean to C14 alkane stress differed markedly from the other three species. Given that Cd shares chemical similarities with mineral elements like Ca and Zn (Huang et al., 2020), the impact of C14 alkane on Cd uptake likely parallels its effects on the absorption of these mineral elements. Meanwhile, results from random forest analysis, mantel tests, and SEM collectively reveals that the phytotoxic effects of C14 alkane are predominantly mediated through the disruption of mineral elements homeostasis, rather than through direct toxicity. Mineral elements play vital roles in all plant life activities (Baxter and Dilkes, 2012). By interfering with the uptake of these essential elements, C14 alkane stress significantly influences plant growth, Cd concentrations, thereby indirectly determining final Cd accumulation levels.

This disruption reveals two distinct plant strategies, distinguished by their ionomic regulatory capacity. Tolerant-regulatory types (ryegrass, castor bean, and mirabilis) exhibited complex internal networks where Cd accumulation was modulated by the interplay of multiple mineral elements (Mn, Fe, K). For example, in castor bean, shoot Fe content positively affected shoot Cd (β = 0.55), revealing a synergistic uptake relationship (Huang et al., 2020). Their tolerance appears linked to an ability to dynamically rebalance these elemental interactions to sustain growth. In stark contrast, the sensitive-direct type (amaranth) displayed a more vulnerable pathway. Here, C14 alkane directly and significantly affects the Cd concentration in roots (β = −0.53) and shoots (β = −0.37), and soil bioavailable Mn directly inhibited biomass (β = −0.47). Concurrently, the significant reduction in shoot Zn concentration under C14 alkane stress represents a crucial factor potentially inhibiting amaranth growth. Random forest analysis identified shoot Zn content as a primary predictor of Cd accumulation in amaranth (Figure 5c), and it was significantly correlated with plant biomass. Zinc participates in various physiological processes, including the repair of the photosystem II complex and mitigation of oxidative stress (Li W. et al., 2025). This pattern reflects a failed ionomic regulation, leaving the plant more exposed to the direct chemical stress of C14 alkane.

Within this framework, Mn emerged as a pivotal and complex mediator, exemplifying the species-specific nature of ionomic management. In the tolerant species ryegrass and castor bean, elevated shoot Mn content was positively correlated with biomass (β = 1.80 and 0.97, respectively), likely by acting as a cofactor for SOD to mitigate oxidative stress (Salomé, 2018). Concurrently, Mn played a key role in Cd competition. In castor bean, increased shoot Mn content was associated with reduced shoot Cd (β = −1.42), illustrating effective competition at transport or sequestration sites (Zeng et al., 2020). Conversely, in ryegrass, shoot Mn positively influenced shoot Cd (β = 1.56), suggesting a co-transport scenario under specific internal thresholds (Ge et al., 2021). In the sensitive species amaranth, soil bioavailable Mn directly inhibited its biomass (β = −0.47). Although C14 alkane stress also significantly increased Mn content in amaranth roots and shoots, the concentration declined at higher stress levels (Supplementary Figures 1i,j). This impaired Mn accumulation response likely contributed to the pronounced growth inhibition observed in amaranth. These findings collectively highlight that Mn exhibits a dual role in regulating both plant growth and Cd translocation, with its net effect being species-specific and dependent on the plant’s overall ionomic management strategy.

This study extends beyond merely documenting the inhibitory effect of organic pollutants on metal uptake. It elucidates the central mechanistic of mineral elements homeostasis in regulating Cd accumulation. We demonstrate that plant species tolerant to petroleum hydrocarbons can potentially mitigate C14 alkane phytotoxicity by regulating the uptake of key mineral elements like Mn. Consequently, interspecific variation in Cd accumulation is not solely a function of direct hydrocarbon tolerance but also reflects the inherent capacity of a species to maintain the balance of critical mineral elements under stress. Finally, our research posits that managing soil nutrient availability—for instance, through targeted micronutrient amendments—could be a viable strategy for enhancing the safe utilization of co-contaminated farmland. Leveraging these mineral elements competition and homeostasis mechanisms could simultaneously alleviate contaminant stress on plants and reduce Cd entry into the food chain.

## 5 Conclusion

This study establishes that the phytotoxicity of C14 alkane and its impact on Cd accumulation are predominantly mediated through alterations in plant mineral elements homeostasis. This “mineral elements-mediated” mechanism serves as a central nexus explaining plant performance in co-contaminated soils. Crucially, plant species exhibited divergent adaptive strategies defined by their ionomic plasticity—the capacity to adjust internal elemental composition under stress. Based on this plasticity, two functional types are identified: tolerant-regulatory species (ryegrass, castor bean, mirabilis), which strategically adjust key mineral balances to cope with stress and influence Cd accumulation; and sensitive-direct species (amaranth), whose failure to regulate ionomic profiles results in severe growth inhibition and dysregulated Cd accumulation. Consequently, our findings propose a paradigm shift: ionomic regulatory capacity, beyond direct contaminant tolerance, is the critical determinant for phytoremediation efficacy in complex contamination scenarios. This provides a predictive criterion for selecting and optimizing plant-based strategies, including targeted mineral elements management, for the remediation of soils co-contaminated with heavy metals and petroleum hydrocarbons.

## Data Availability

The raw data supporting the conclusions of this article will be made available by the authors, without undue reservation.
