# Supplementary material for: Mineral elements-mediated responses govern cadmium accumulation in plants under C14 alkane stress
Source: Front Microbiol. 2026 Jun 1;17:1830534. doi: 10.3389/fmicb.2026.1830534 (PMC13265540; doi:10.3389/fmicb.2026.1830534)
Supplement: Supplementary file 1 [file Supplementary_file_1.docx]

**Supplementary Materials to** “Mineral elements-mediated responses govern cadmium accumulation in plants under C14 alkane stress”

Lizhu Yuan^a*^, Xinzhuo Qian^b^, Weijin Zheng^a^ , Xu Chen^a^, Boxi Lv^a^, Xuemei Zhong^b^, Jonathan W.C. Wong^a*^

^a^ Research Center for Eco-Environmental Engineering, Dongguan University of Technology, Dongguan 523808, China

^b^ College of Earth Sciences, Guilin University of Technology, Guilin 541004, China

^*^ Corresponding author.

E-mail address: [2022019@dgut.edu.cn](mailto:2022019@dgut.edu.cn) (LZ Yuan), [jcwong@associate.hkbu.edu.hk](mailto:jcwong@associate.hkbu.edu.hk) (Jonathan W.C. Wong).

**Number of Pages: 4**

**Number of Figures: 1**

**Number of Tables: 1**

**Table S1** The properties of the clean soil.

| Characteristics | Value |
| --- | --- |
| Particle size analysis (%) |  |
| Sand (%) | 21.56 |
| Silt (%) | 39.78 |
| Clay (%) | 38.66 |
| Organic matter (%) | 1.57 |
| CEC (cmol/kg) | 14.28 |
| pH | 7.08 |
| Electrical conductivity (mS·cm^-1^) | 0.278 |


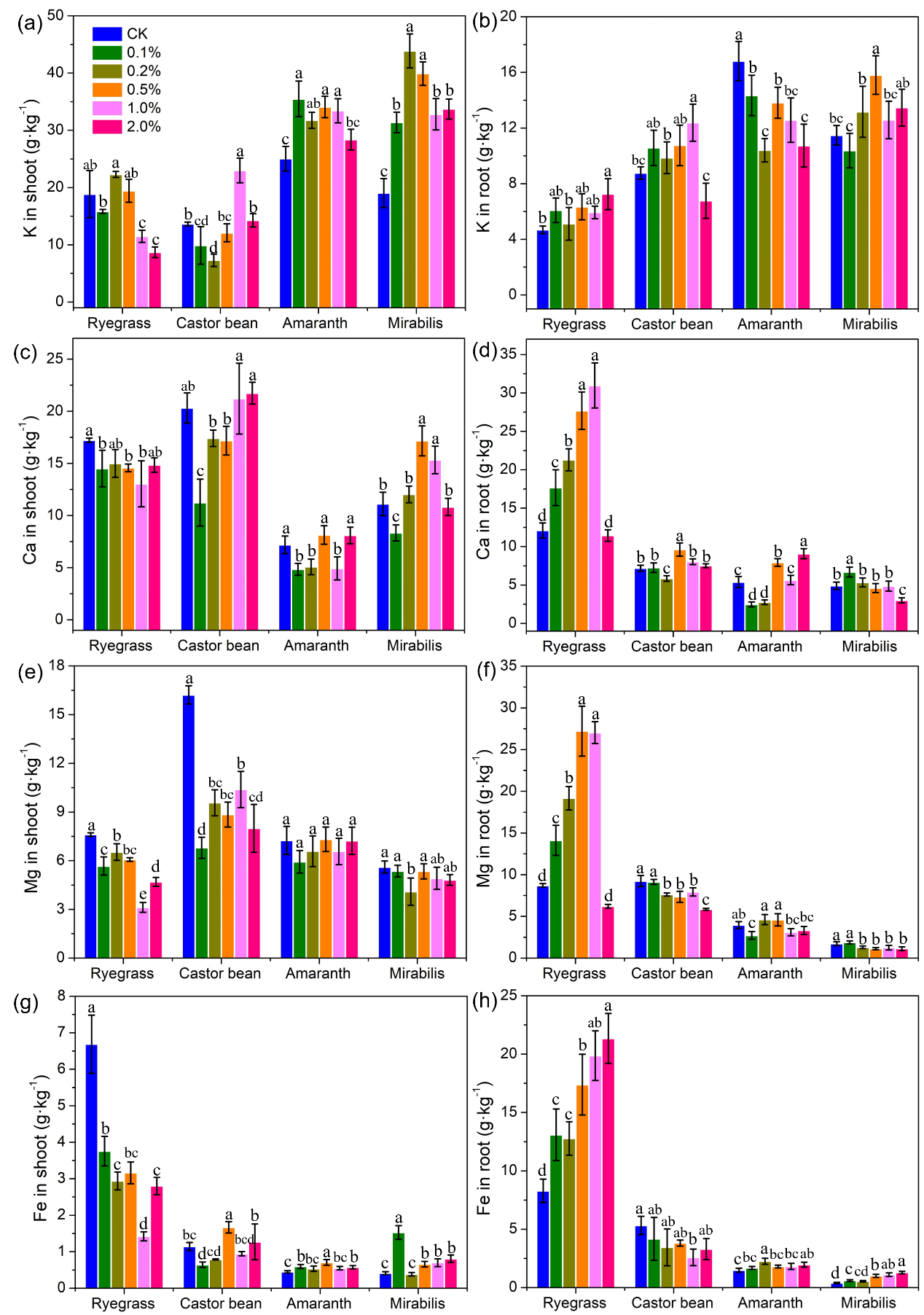


**Fig. S1.** Changing mineral elements (K, Ca, Mg, Fe, Mn, Cu, and Zn) in shoots and roots of different plants in response to C14 alkane stress. (a) and (b), (c) and (d), (e) and (f), (g) and (h), (i) and (j), (k) and (l), (m) and (n) represent K, Ca, Mg, Fe, Mn, Cu, and Zn content in the shoots and roots of different plants, respectively. Each value represents the mean ± standard deviation of three independent experiments. Different uppercase letters on the column represented significant difference in different treatments of C14 alkane concentration (*P*<0.05).


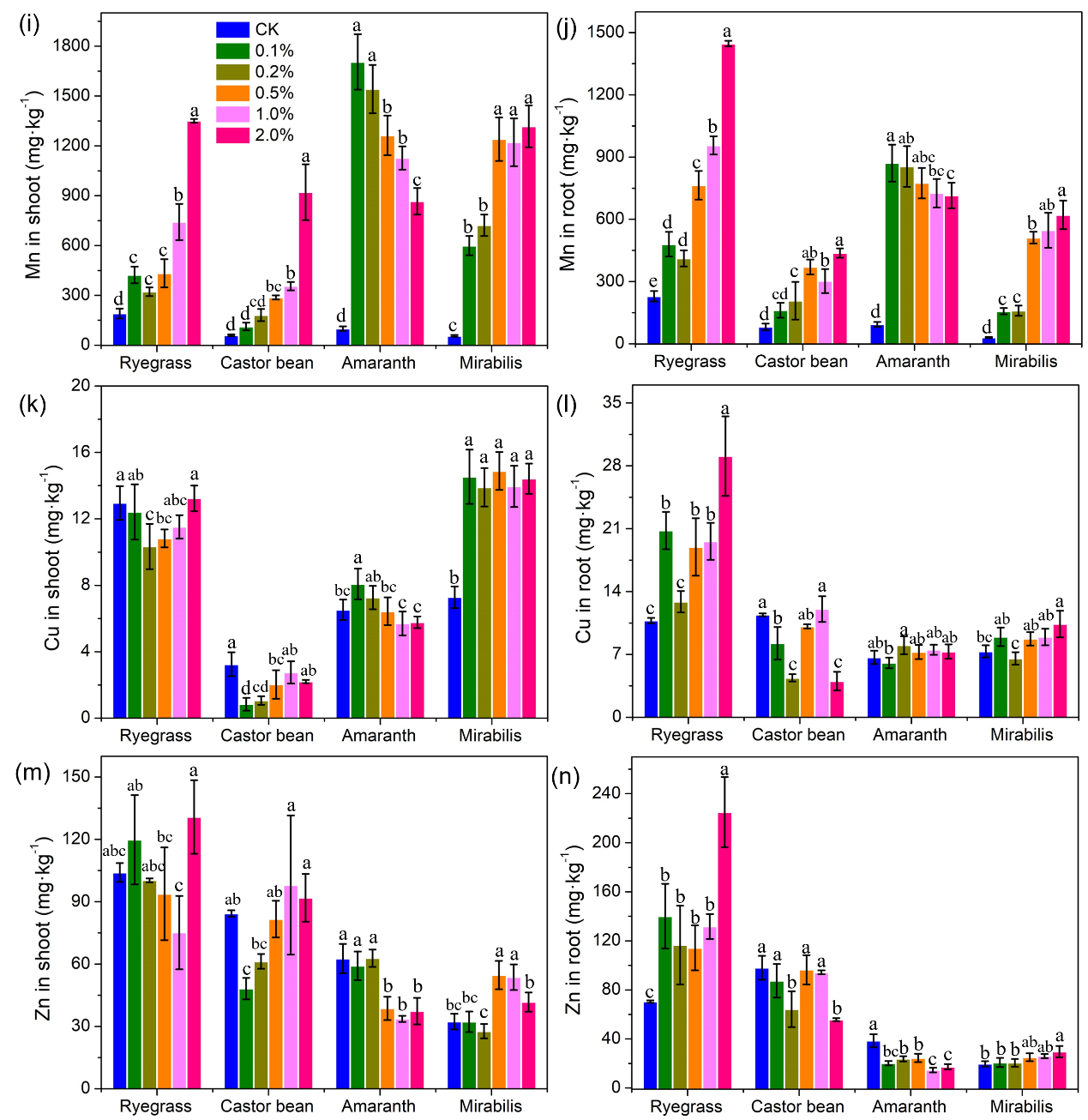


**Fig. S1.** (continued)
